# Granulomatosis With Polyangiitis Mimicking Giant Cell Arteritis

**DOI:** 10.7759/cureus.78502

**Published:** 2025-02-04

**Authors:** Abdullah Motam, Aemen Khalid, Mohamed Elhadi

**Affiliations:** 1 Internal Medicine, Royal Blackburn Hospital, Blackburn, GBR; 2 Internal Medicine, Royal Preston Hospital, Preston, GBR

**Keywords:** acute sinusitis, avacopan, cranial arteritis, granulomatosis with polyangiitis (gpa), pulsed-dose steroids, pyrexia of unknown origin (puo), rituximab, wegener’s granulomatosis

## Abstract

Granulomatosis with polyangiitis (GPA) is a rare auto-immune ANCA-associated small-vessel vasculitis characterized by necrotizing granulomatous inflammation, primarily affecting the sinuses, respiratory tract, and kidneys. Early diagnosis and treatment are crucial for improving patient outcomes and preventing rapidly progressive renal failure. We present a case of a 66-year-old woman presenting to the emergency department (ED) with a two-week history of productive cough, nausea, fevers, and headache. Observations and routine blood tests were unremarkable, so the patient was treated with antibiotics for a chest infection. Headache became a particularly prominent feature, along with jaw claudication and temporal tenderness. Treatment for another vasculitis, giant cell arteritis (GCA), was started and then stopped after ultrasound scanning showed no signs of GCA. After repeated admissions to the hospital, she was eventually diagnosed with GPA after the auto-immune screening was sent, which was positive for cANCA (anti-PR3). This case highlights a high degree of diagnostic uncertainty due to multiple investigations revealing the involvement of different organs, but these were treated separately. A more holistic approach to multi-system pathology, as well as considering other causes of fever and raised inflammatory markers, would have spared the patient from many unnecessary and invasive investigations. The patient eventually made a rapid recovery after the initiation of appropriate immunosuppressant therapy.

## Introduction

Granulomatosis with polyangiitis (GPA), formerly known as Wegener's Granulomatosis, is a rare auto-immune ANCA-associated small-vessel vasculitis affecting multiple organs. It is characterized by necrotizing granulomatous inflammation throughout systemic vasculature, primarily affecting the sinuses, respiratory tract, and kidneys. Early diagnosis and treatment are crucial for improving patient outcomes and preventing rapidly progressive renal failure [1]. Unfortunately in this case, a high degree of diagnostic uncertainty not only led to delays in inducing remission, it also led to significant reduction of quality of life for the patient over several weeks, and unnecessary invasive investigations. Headache, jaw claudication, and temporal tenderness, symptoms usually associated with giant cell arteritis (GCA), a large-vessel vasculitis, led to further diagnostic dilemmas. It is important to note, as in this case, renal involvement may only be a late manifestation, and should not reduce your index of suspicion of GPA in the presence of sinonasal and pulmonary symptoms. 

## Case presentation

**Figure 1:**
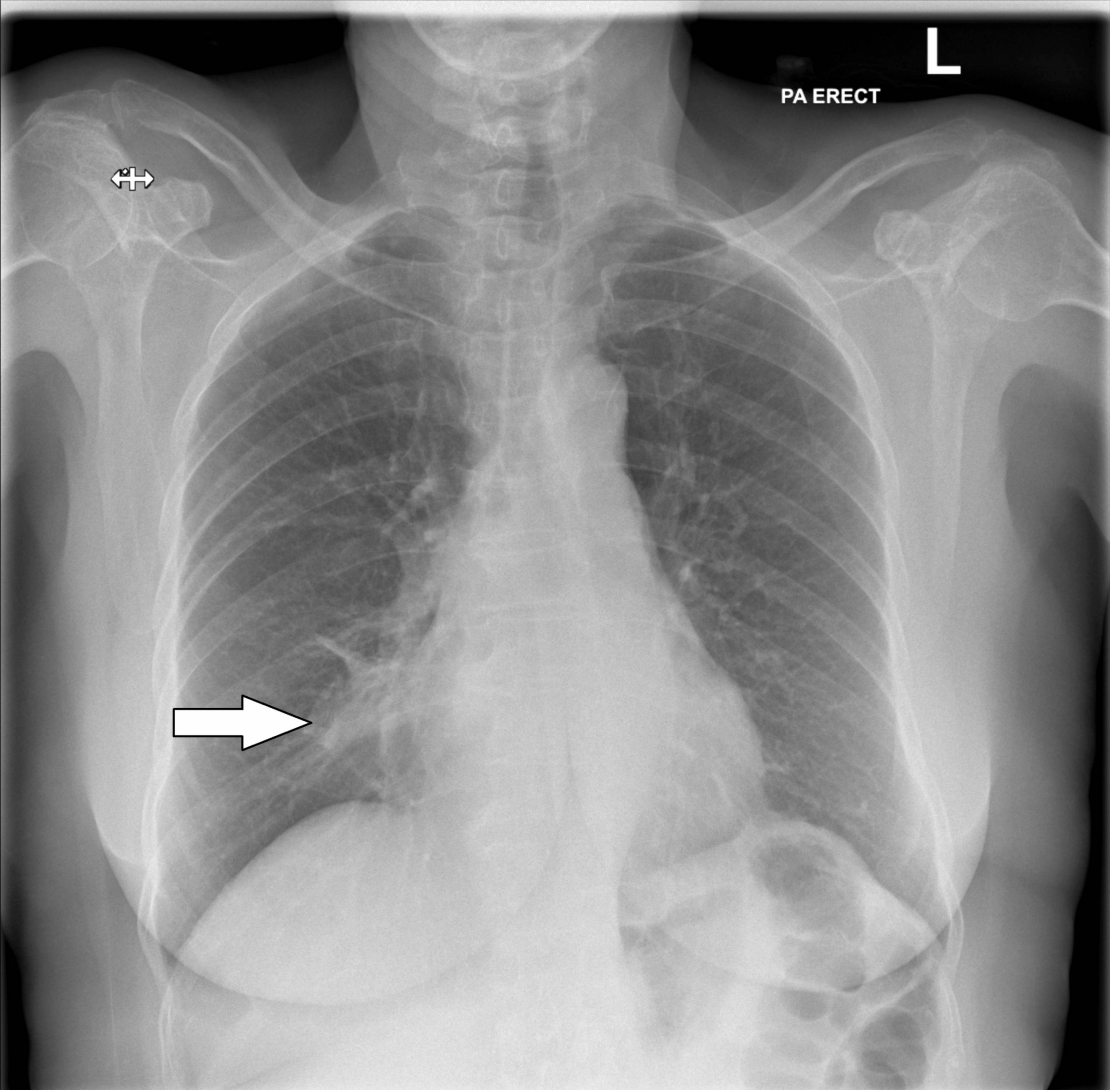
Initial CXR showing right middle-lobe consolidation CXR: chest x-ray

A 66-year-old woman presented to the emergency department (ED) with a two-week history of productive cough, nausea, fevers, and headache. Observations and blood tests (renal function, full blood count) were unremarkable, except for a C-reactive protein (CRP) level of 74 mg/L (normal range: 0-5 mg/L). A chest X-ray (CXR) (Figure 1), was also performed; however, the consolidation was unfortunately missed, and she was discharged with oral antibiotics (a five-day course of amoxicillin), to treat bronchitis.

**Figure 2:**
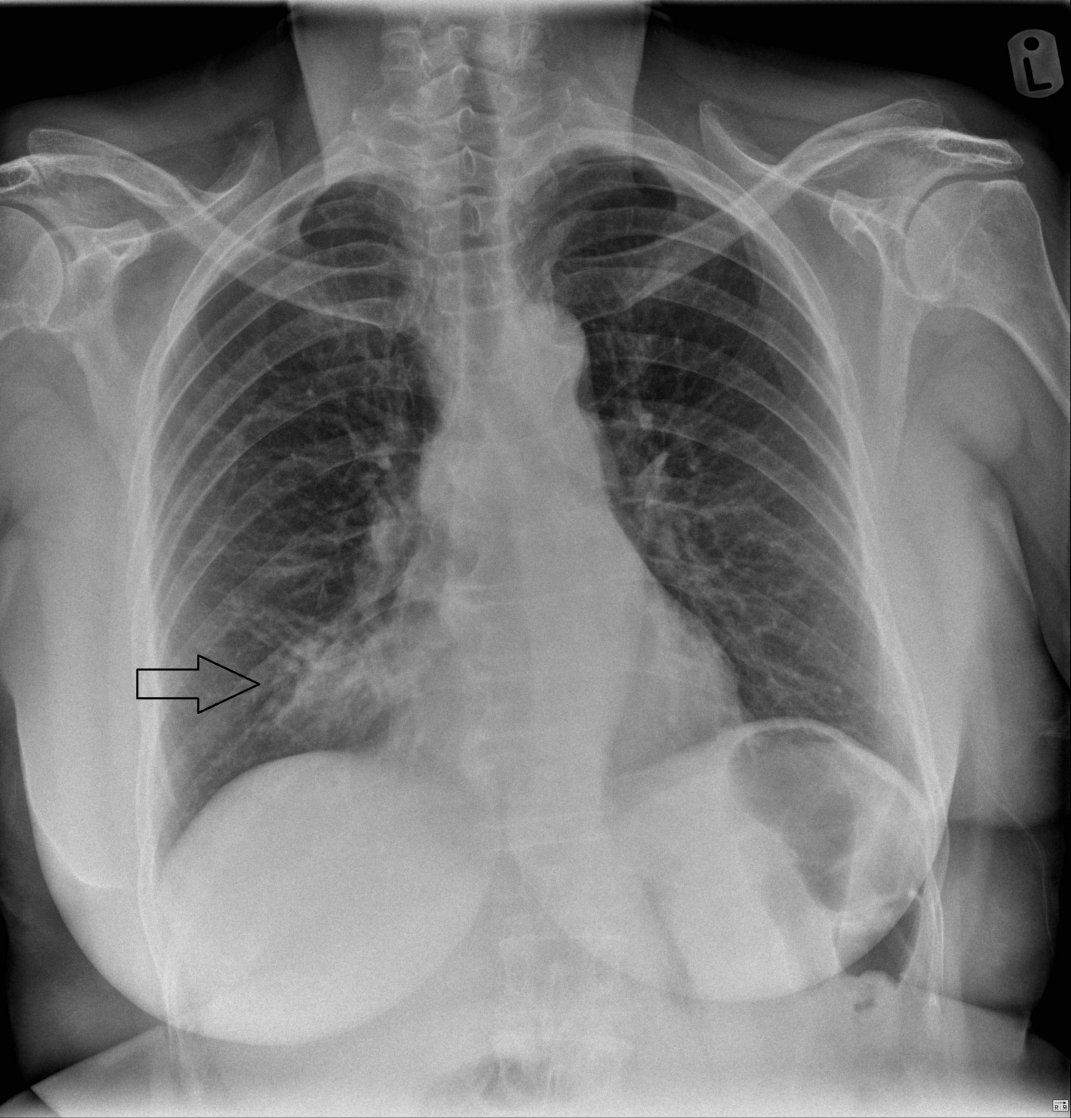
Progression of the previously demonstrated consolidation. This CXR was performed 10 days later CXR: chest x-ray

She proceeded to attend the ED on three more occasions, with progressively worsening symptoms, including the development of hemoptysis, severe headache, and malaise, despite three further courses of antibiotics (given further five-day courses of clarithromycin, doxycycline, and co-amoxiclav) treating for pneumonia, after serial CXRs revealed a refractory area of dense consolidation in the right middle lobe (Figure 2). She was eventually admitted and investigated for GCA due to the temporal nature of the headache, temporal tenderness, and jaw pain, especially during mastication. She was started prophylactically on high-dose steroids (40 mg prednisolone), and subsequently, ultrasound Doppler scanning of the temporal arteries bilaterally revealed no evidence of GCA. No temporal biopsy was performed. It was noted that she felt much better on the steroids; however, these were discontinued a few days after commencement, after the scan was negative. She was reviewed by the ENT team, who diagnosed acute non-infective sinusitis, and she was discharged with saline, xylometazoline, and betamethasone nasal drops.

She re-attended the hospital with worsening symptoms, the most prominent being a crushing headache, ongoing sinusitis, and rigors. Observations revealed a high-grade pyrexia of 39.5°C, but otherwise unremarkable. Physical examination was unremarkable. Blood tests revealed raised inflammatory markers, but normal kidney function (Table 1). Multiple sets of blood, sputum, and urine cultures were negative. Atypical pneumonia (pneumococcal and legionella urinary antigens, mycoplasma PCR), viral, and TB screening were negative. Beta-D-glucan and galactomannan tests were sent to investigate possible fungal infection; these were also negative.

**Table 1:** Laboratory results WCC: white cell count; CRP: C-reactive protein; ESR: erythrocyte sedimentation rate

Laboratory test	On admission	Peak	Reference range
WCC	10.1	22.1	4.0-10.9 × 10^9^/L
CRP	76	299	0-5 mg/L
ESR	72	>96.0	0-10 mm/hr
Hemoglobin	125	88 (trough)	120-150 g/L
Platelets	459	798	150-450 × 10^9^/L
Creatinine	70	70	45-84 μmol/L

**Figure 3:**
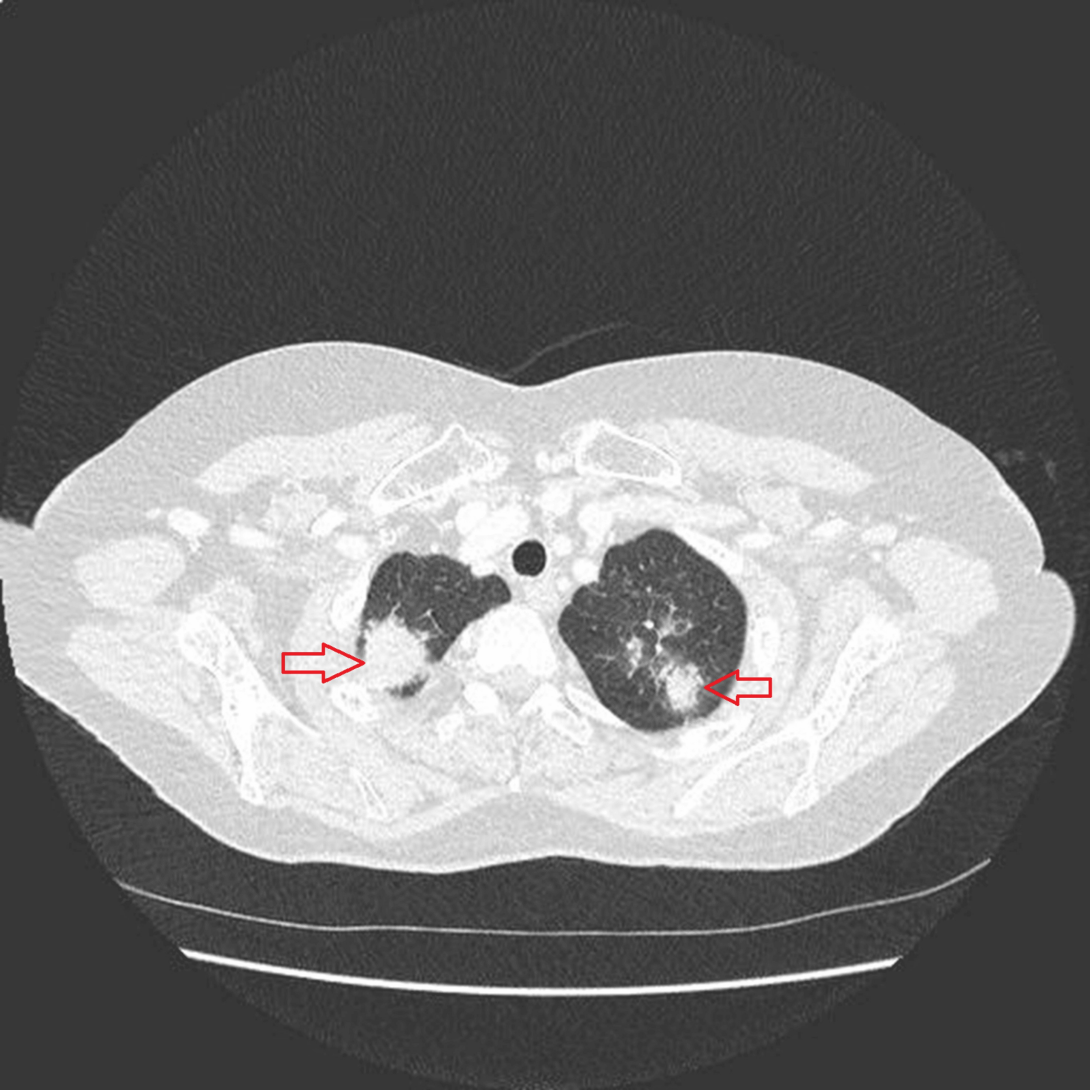
CT scan showing bilateral apical opacification

CXR showed right middle-lobe consolidation. A CT scan of the thorax with contrast (Figure 3) revealed bilateral dense mass-like consolidation in the lung apices, which was thought to be TB, as well as right middle-lobe consolidation and atelectasis. She was isolated in a cubicle, and bronchoscopy was performed for bronchoalveolar lavage, all samples taken were negative for TB infection. MR imaging of the head revealed significant high-signal changes in the cranial vessels signifying cranial arteritis. After several courses of escalating antibiotics, including eight days of meropenem, which did not improve the patient's condition clinically or biochemically, and following consultation with the microbiologist, the decision was made to discontinue antibiotics, and repeat cultures were sent after 48 hours.

Due to the marked nature of the deterioration of the patient's condition, headaches, rising inflammatory markers, and ongoing fevers, auto-immune screening was sent. This came back with a strongly-positive titer of ANCA against PR3 (143.5 IU/mL, normal range 0-1.9 IU/mL). 

She was referred to the rheumatology team who diagnosed the patient with GPA affecting the sinuses, lungs, and cranial vessels (by causing inflammation of the vasa vasorum). The patient was swiftly initiated on avacopan and high-dose steroids to induce remission, and then subsequently treated with a maintenance infusion of rituximab.

## Discussion

GPA is a vasculitis disease that can affect vessels in any organ, including the brain. The multisystemic nature of the condition means that it has a wide variety of presentations and symptoms, which can lead to diagnostic uncertainty. Often the initial presentation may only be single-organ involvement, such as in this case, where it was primarily a productive cough indicating a pulmonary problem. However, as the case developed and certain symptoms became more prominent (notably the headache, malaise, fevers, and night sweats), suspicion of something more sinister, such as TB infection and malignancy began to take hold.

The finding of cranial arteritis was concerning as the clinical picture did not fit with GCA, which typically presents with a unilateral, temporal headache with jaw claudication, and tenderness. The explanation: GPA can cause vasculitis, even of the vasa vasorum of larger vessels, which would not classically be considered "small vessels." In this way, GPA can affect large vessels as well as smaller vessels, and mimic other types of vasculitides, causing an "overlap syndrome" [2].

Treatment with steroids to induce remission was very successful, with the patient feeling better almost immediately after treatment initiation. The strategy employed to start avacopan as a steroid-sparing medication was decided as the patient was elderly, with a high risk of potential side effects from high-dose steroids. Avacopan is an orally administered C5a receptor antagonist proven to show non-inferiority to steroids in clinical trials comparing treatment efficacy in microscopic polyangiitis (MPA) and GPA [3]. The patient was still protected from steroid side effects by the initiation of a proton pump inhibitor, osteoporosis prophylaxis (vitamin D-calcium supplementation), fluconazole, and cotrimoxazole (to protect against opportunistic infections).

Rituximab is a well-established treatment used to maintain remission in patients with MPA and GPA [4]. It is an anti‐CD20 monoclonal antibody that targets and depletes CD20+ B cells, which produce autoantibodies causing inflammation such as vasculitis. On reviewing the literature, it is well documented that rituximab has been used for years to treat ANCA-associated vasculitides with remission rates up to 93%, the five-year survival was 74-91%, and relapse (variably defined) was common in the first two years but the frequency varied; 18% to 60% in GPA cases [5].

## Conclusions

GPA is an auto-immune vasculitis primarily affecting small vessels and multiple organs. It has a particular predisposition to affect the sinonasal, pulmonary, and renal vasculature. A triad of cough, sinusitis, and AKI is a common presentation; however, one or more of these may often be absent. Furthermore, due to the nature of the disease and its ability to affect any vessel in any organ, insidious and unexpected presentations occur frequently. The disease process can mimic infectious and malignant pathologies, and, as demonstrated in this case, other vasculitides, which can delay accurate diagnosis and treatment, causing significant diagnostic uncertainty.

The gold standard test for diagnosis is an auto-immune screen, primarily focusing on ANCA. Anti-PR3 antibodies are most specific to GPA, and the clinical picture usually helps confirm the diagnosis. In cases where uncertainty remains, a biopsy may be sought. Treatment focuses on inducing remission with glucocorticoids and steroid-sparing immunosuppressants, such as avacopan. Remission is maintained with immunosuppressive therapy, primarily rituximab or cyclophosphamide.
